# An 11-year prospective study of personality X parenting interactions as predictors of self-efficacy in young adults: diathesis-stress or differential susceptibility?

**DOI:** 10.1186/s40359-021-00676-6

**Published:** 2021-11-03

**Authors:** Marike H. F. Deutz, Willemijn M. van Eldik, Vera T. Over de Vest, Ank Ringoot, Amaranta D. de Haan, Peter Prinzie

**Affiliations:** 1 Department of Psychology, Education and Child Studies, Erasmus School of Social and Behavioural Sciences, Erasmus University Rotterdam, PO Box 1738, 3000 DR Rotterdam, The Netherlands; 2 Present Address: HYPE Centre of Expertise on Early Intervention for Borderline Personality Disorder, GGz Centraal, Amersfoort, The Netherlands; 3 Present Address: Youz, Parnassia Psychiatric Institution, The Hague, The Netherlands

## Abstract

**Background:**

Self-efficacy, individuals’ beliefs regarding their capacities to perform actions or control (potentially stressful or novel) events, is thought to be important for various life domains. Little however is known about its early precursors. This study examined the predictive effects of childhood personality and parental behaviors (i.e., overreactive discipline and warmth) for general self-efficacy in young adulthood. Furthermore, it was examined whether personality and parenting behaviors interacted and whether these interactions supported the diathesis-stress or differential susceptibility model. These aims were examined in an 11-year prospective study of 336 participants (*M*_age_ at T1 = 10.83 years, *range* = 9–12 years, 53.9% girls). Personality and parental behaviors were reported at T1 by both mothers and fathers, whereas self-efficacy was self-reported at T2 11 years later. Hypotheses were tested in M*plus* using multilevel structural equation modeling.

**Results:**

Results revealed that (only) emotional stability, and not parenting, predicted higher self-efficacy 11 years later. Benevolence functioned as a susceptibility marker in the association between overreactivity and self-efficacy.

**Conclusions:**

The results show that childhood emotional stability is an important long-term predictor of self-efficacy, even into emerging adulthood. Moreover, the integration of individual differences in models of parenting effects may further improve our understanding of early adults’ adjustment.

## Introduction

Self-efficacy is a key construct in multiple fields of psychology, including developmental, health and personality psychology [1, 2]. It refers to individuals’ beliefs regarding their capacities to perform actions or control (potentially stressful or novel) events that impact their lives [2, 3]. Self-efficacy is thought to be an important aspect of functioning in various life domains such as academic achievement, life satisfaction, mental well-being, and a successful school-to-work transition [2, 4–7]. Although self-efficacy can be task- or domain-specific (e.g., academic self-efficacy), it can also be conceptualized as a broad and stable sense of individuals’ confidence in capabilities to master challenges across a wide range of demanding or novel situations and is then commonly referred to as ‘general self-efficacy’ [1, 2]. Despite the importance of self-efficacy for general functioning, relatively little is known about individual and proximal contextual factors that affect its development.

According to Bandura’s self-efficacy theory [3], self-efficacy is influenced by interactions between individual and environmental factors. Empirical evidence of prospective relations among individual and environmental factors and self-efficacy however is scarce. Therefore, the current study has the following two (overarching) research aims. First, we aim to examine prospective relations between personality and parental behaviors in childhood (9–12 years) and self-efficacy 11 years later in early adulthood. Personality was selected as it is considered a key construct to describe relatively stable individual differences [8, 9]. Warmth and overreactive parenting were selected as these are two key parental behaviors that fall into the dimensions of support and control respectively [10, 11], and which, moreover, are potentially malleable environmental factors. Second, we aimed to examine whether personality moderates associations between childhood parenting behaviors and early adult self-efficacy. Specifically, we empirically tested whether the diathesis-stress (i.e., certain personality traits are linked to vulnerability and later problems in adverse environments [12]) or the differential susceptibility [13] model of person-environment interaction (i.e., these personality traits are susceptibility factors, predicting problems in adverse environments but *also* better outcomes in good environments) was supported.

### Parenting and self-efficacy

Parents are thought to play an important role in self-efficacy, even when the child is an emerging adult and might no longer reside with his or her parents (see e.g., [14]). Emerging adulthood is a time in which great (potential stressful) changes in autonomy development occur and other important social contexts, such as the peer group, take a more eminent place in the individual’s life [15]. The self-determination theoretical framework [16], which is closely aligned with self-efficacy theory [17], is useful in describing how parenting can affect self-efficacy. According to the self-determination theory, all individuals have basic psychological needs for autonomy, relatedness, and competence, of which fulfillment is essential to well-being. Self-efficacy is often used as a proxy for competence as these concepts are thought to be intertwined [15, 17]. Whereas all needs can be either promoted or hampered by environmental factors, competence is thought to be a direct outcome of parenting behaviors [15].

Parenting behaviors are often classified under two broad parenting dimensions characterizing the quality of the parent–child interaction (i.e., warmth, support) and the nature of parental discipline (control) [10, 18]. In the current study, we focus on warmth (i.e., support) and overreactive (i.e., controlling) parenting as two key parenting behaviors that are both expected to impact self-efficacy [19]. Warmth refers to behaviors of acceptance, nurturance and affection [20] and is generally recognized as a central influence in early socialization [21]. If parents display warm and supportive behavior towards their child, this gives the child a secure base from which exploration can occur, and to which the child can return when challenging situations come about. Warmth is thus thought to foster autonomy development and, in turn, competence/self-efficacy [22, 23]. Overreactive parenting, on the other hand, refers to controlling, aversive, and intrusive reactions to child problematic behaviors [21, 24]. When parents show overreactive discipline, this might hinder self-efficacy development, since such parenting behavior restrict the child’s autonomy, which can lead to doubts about one’s feelings of competence [23]. Such parenting behaviors may ultimately lead to children fearing to do something wrong, and therefore potentially missing important learning experiences in which they could have experienced feelings of competence [23].

Several cross-sectional studies in diverse samples have demonstrated links between parenting behaviors and general self-efficacy in childhood, adolescence and young adulthood. These studies showed relations between lower general self-efficacy and lower support or autonomy-supportive parenting [15, 23, 25], higher psychological control [23, 25], and more helicopter, or (over-)controlling parenting [15, 26–28]. As most of these studies were conducted in specific populations such as college students [15, 26–28], Iranian-American adolescents [23], or immigrant families [25], results cannot be generalized to the (Western) population. For example, in Iranian adolescents authoritarian paternal parenting was unexpectedly positively related to general self-efficacy, which the authors explain with the collectivist culture in Iran where authoritarian parenting is not necessarily considered an ineffective parenting style [29].

Unfortunately, prospective research on links between parenting behaviors and general self-efficacy is scarce, and especially research regarding the long-term impact of parenting on young adults’ general self-efficacy is lacking. As parenting behaviors are partly malleable, this knowledge could have implications for intervention programs focusing on strengthening young adults’ well-being, including general self-efficacy. In this study, we therefore examined prospective associations between two important parenting behaviors, warmth and overreactive discipline, and general self-efficacy in young adulthood.

### Diathesis-stress and the differential susceptibility model

Personality, which refers to tendencies to think, feel and behave in certain consistent ways [30], is a key individual factor affecting functioning and has been shown to be related to (general) self-efficacy. Although mostly examined in adult samples and at one time point, (G)SE appears to be related to personality, especially to the traits of (lower) neuroticism, and (higher) extraversion and conscientiousness (e.g., [31–34]). With regard to children, highly conscientious children are thought to have more willingness to work hard, are better able to focus on goals and hold greater beliefs that they can achieve desired outcomes, increasing self-efficacy. Children higher in neuroticism are more emotionally unstable, less confident and have more trouble making decisions. They are often more anxious, which can suppress or decrease self-efficacy [35]. More extraverted children are thought to show more initiative and assertiveness and more general confidence in their abilities, and they also elicit more positive responses from others, which can increase self-efficacy [33, 34].

Moreover, in addition to these direct associations, ecological transactional models emphasize that integrating parent and child effects into one theoretical model may better explain heterogeneity in adjustment than a consideration of these effects in isolation [36]. Personality is thought to be an important individual factor that shapes individual’s sensitivity to environmental influences. Studies examining personality as a moderator of environmental factors on individual outcomes, have shown for example that personality and parenting interact in predicting social adjustment in school [37], aggression and delinquency [38] as well as anxious and depressive problems [39]. The role of parenting in young adult self-efficacy might thus differ depending on children’s personality traits, which can be described with two influential models of person-environment interactions: the diathesis-stress model and the differential susceptibility model. The diathesis-stress model states that children that posit certain individual factors, such as personality traits, are more vulnerable as they show worse outcomes in more adverse environments [12, 40]. The differential susceptibility expands this view, because it adds a positive notion and pinpoints the advantages of certain traits in the context of supportive environments [13, 41]. The differential susceptibility model posits that children with certain individual traits not only show worse outcomes in more adverse environments, but also better outcomes in more optimal environments. They are thus not vulnerable, but more susceptible to the environment than others, as they are affected by the environment both for *better* and for *worse* [40].

### The current study

Summarizing, the current study aims to examine relations between parenting in childhood and child personality and self-efficacy in young adulthood, as well as the potential moderating role of personality in associations. We add to existing knowledge by employing a prospective design spanning 11 years, thereby extending previous research on parenting and self-efficacy that is mostly cross-sectional. Mothers and fathers reported on their child’s personality and their own warmth and overreactive discipline when the child was aged 9–12 years, and self-efficacy was self-reported 11 years later during early adulthood (20–23 years). Based on the literature, our hypotheses are that [1] parenting behaviors, specifically less warmth and more overreactive discipline, predict lower self-efficacy (e.g., [22, 23]), (2) personality, especially higher neuroticism and lower extraversion and conscientiousness predict lower self-efficacy (e.g., [31–34]), and (3) that associations between parenting behaviors and self-efficacy would depend on child personality traits, either for children with a more vulnerable personality profile (*diathesis-stress*) or ‘for better and for worse’ (*differential susceptibility*). Specifically, according to both the diathesis-stress and the differential susceptibility models, low warmth and high overreactive discipline (reflecting a more adverse environment) would be associated with lower young adult self-efficacy for children with less optimal personality traits. The differential susceptibility model additionally states that high warmth and low overreactive discipline would also be associated with higher young adult self-efficacy for children with less optimal personality traits.

By examining if certain personality traits make children either more vulnerable or more susceptible to parental influences on self-efficacy, we extend knowledge on how self-efficacy is shaped. Examining both models statistically can lead to more fine-grained knowledge on associations between parenting, personality and self-efficacy and more refined implications for promoting self-efficacy. Ultimately, this knowledge could aid in potentially identifying children that could benefit from targeted interventions aimed at boosting self-efficacy, which in turn could positively affect functioning across different domains.

## Method

### Participants and procedure

This study is part of the ongoing longitudinal Flemish Study on Parenting, Personality, and Development (FSPPD; e.g. [42]). In 1999, a proportional stratified sample of elementary-school-aged children attending regular schools in Flanders (Belgium) was randomly selected. Strata were constructed according to geographical location (i.e., province), age and gender. All participants provided written informed consent and all procedures were approved by the board of the KU Leuven. At the first assessment in 1999, 674 families participated of which 92.5% were two-parent families, 50% of the children were boys, and all children had the Belgian nationality. In this study, data of the 4th (T1: 2004) and 8th wave (T2: 2015) were used because these waves contained the measures of interest. At T1, families received questionnaires per mail and at T2 questionnaires were distributed online. We included all participants with data of at least one informant on personality and parenting at T1 and complete data at T2 for self-reported self-efficacy. This resulted in a sample of *N* = 336 children (46.1% boys and 53.9% girls). The recruited sample initially committed to a four-year study and over the 16-years span of the main reasons for dropping out were (1) no longer willing to participate and (2) loss of contact information, mainly during the transition to emerging adulthood including moving to independent homes.

At Time 1, we used both parents (if available) as informants to measure personality and parenting. Self-efficacy was measured using self-report at Time 2. At Time 1, mean age of the children was *M* = 10.83 years (range 9.00–12.92 years). The mean age of the parents was *M* = 40.20 years (range 30.08–54.92) for mothers and 42.01 years (range 31.67–63.67) for fathers. At Time 2, 11 years later, the participants were aged 20.00–23.92 years and the mean age was 21.83 years. Percentages of various educational levels were as follows for mothers and fathers, respectively: elementary school (0.6%, 2.4%), secondary education (33.0%, 39.6%), non-university higher education (50.9%, 35.4%), university (14.6%, 20.5%), missing (0.9%, 2.1%). At T2, 62% of the emerging adults lived with their parents, 23.1% lived in a shared student home, 9.9% had an independent living situation, and 4.8% described their situation as different.

Missing data analysis was conducted,^1^ showing that participants who participated at both T1 and T2 (*N* = 336) and participants who only participated at T1 (*N* = 176) did not differ statistically from each other on age or measures of parenting and personality, except for father reported extraversion (*M*_incomplete_ = 3.24 (*SD* = 0.33), *M*_complete_ = 3.16 (*SD* = 0.33), *t* (462) = 2.56, *p* = 0.013) and mother reported imagination *M*_incomplete_ = 3.35 (*SD* = 0.46), *M*_complete_ = 3.45 (*SD* = 0.45) (*t* (465) = − 2.21, *p* = 0.027). Little’s missing completely at random test indicated that data were missing completely at random χ^2^ (474) = 451.83, *p* = 0.761.

### Measures

#### Parental overreactivity

Maternal and paternal overreactive discipline was measured at T1 using parents’ self-reports of the Dutch translation of the Parenting Scale [43, 44]. The Overreactivity scale consists of nine items and assesses angry, frustrated, and aversive reactions to their child’s problematic behavior. Items are formulated as hypothetical situations of discipline encounters (e.g., “When my child misbehaves…”) followed by two opposite anchor points rated on a 7-point Likert scale (e.g., ‘I speak to my child calmly’—‘I raise my voice or yell’). Mean scores were computed. Cronbach’s alphas were .77 for mother-reports and .76 for father-reports. Mother- and father-reports correlated weakly (*r* = .26).

#### Parental warmth

Maternal and paternal warmth were measured at T1 using parents’ self-reports of the Parenting Practices Questionnaire [PPQ; [45]). The Warmth scale consists of 11 items and assesses the extent to which parents are affectionate to their child and are involved in their child’s life (e.g., ‘I express affection by hugging, kissing, and holding my child’). All items are rated on a five-point Likert scale ranging from 1 (*never*) to 5 (*always*). Mean scores were computed. Cronbach’s alphas were .82 for mother-reports and .85 for father-reports. Mother- and father-reports correlated weakly (*r* = .34).

#### Children’s big five personality

Children’s personality dimensions were measured at T1 using mother- and father-reports of the Hierarchical Personality Inventory for Children (H*i*PIC; [46]). The H*i*PIC consists of 144 items and assesses 18 facets of 8 items that are grouped under five higher order domains: (1) Extraversion (32 items, e.g., ‘has an excess of energy’); (2) Benevolence (40 items, e.g., ‘obeys without protest’); (3) Conscientiousness (32 items, e.g., ‘Likes to have things in order’); (4) Emotional Stability (16 items, e.g., ‘is quick to worry about things (reversed)’), and (5) Imagination (24 items, ‘Likes learning new things’). All items are rated on a five-point Likert scale ranging from 1 (*barely characteristic*) to 5 (*highly characteristic*). Cronbach’s alphas for the mother- and father-reports ranged from .89 for father reported emotional stability to .94 for mother and father-reported conscientiousness, (mean *α* = .92). For every dimension, scores were averaged across items. Correlations between mother and father reports were high for all five factors (*r*_*average*_ = .70, range = .67 (Benevolence, Emotional Stability and Imagination) to .79 (Conscientiousness)). Therefore, scores of mother and father reports were averaged.

#### Self-efficacy

Self-efficacy was measured at T2, using young adults’ self-reports of the Dutch translation of the Generalized Self-Efficacy Scale [47, 48]. This questionnaire consists of 10 statements and assesses the extent to which an individual has optimistic self-beliefs to cope with stressors and difficult life situations (e.g., ‘I always manage to solve difficult problems, if I put enough effort into it’). All items are rated on a four-point Likert scale, ranging from 1 (*not at all true*) to 4 (*exactly true*). A mean score was computed. Cronbach’s alpha in the current study was .84.

### Statistical analyses

**Fig. 1:**
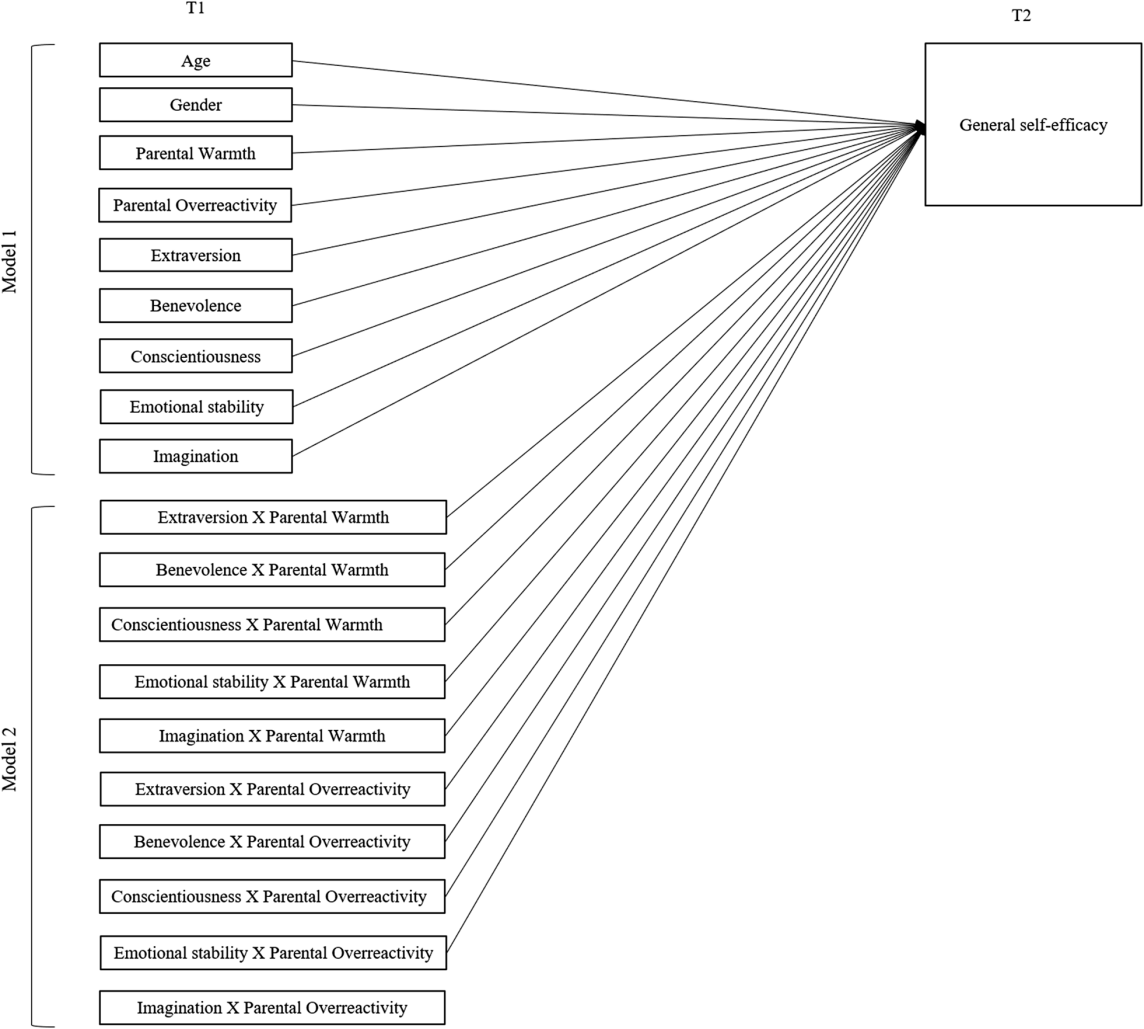
Graphic presentation of the overall statistical model. *Note.* This model was assessed in a multi-group model with a mother-model and a father-model, including respectively maternal warmth and overreactivity versus paternal warmth and overreactivity

First, descriptive statistics and bivariate correlations among all variables were calculated. Then, linear regression analyses were conducted using M*plus* 8.3 [49]. Age and gender of the child, and the main effects of parental warmth and overreactivity and the Big Five personality dimensions were included in the first model. The ten interaction terms of parenting with the Big Five dimensions were added in the second model (see Fig. 1). To reduce multicollinearity as well as to facilitate the interpretation of interaction effects, predictor and moderator variables were standardized before the interaction terms were computed [50]. Maximum likelihood with robust standard errors (MLR) estimation was used to account for potential nonnormality when estimating standard errors [51] and full information likelihood (FIML) was used to take into account missing data. Dependence of informants was accounted for by adjusting standard errors using a sandwich estimator by specifying the *complex* option in M*plus* 8.3 [52].

To interpret significant interaction effects in terms of differential susceptibility or diathesis-stress, we conducted five post hoc analyses according to statistical recommendations outlined by Roisman et al., [53]. First, the effect of parenting was plotted as a function of the moderator (i.e., personality factor) using the Johnson-Neyman technique (J-N technique; [54, 55]) to reveal the regions of significance (RoS) of personality wherein the relationship between parenting and self-efficacy was significant. The region of significance was determined by the 95% confidence interval. If the 95% confidence interval contained the value of zero, then the association between parenting and self-efficacy was not significant. The changes in magnitudes of the slope of parenting on self-efficacy are also presented in the plot for regions of significance [56]. Second, simple slopes analyses were used to estimate the magnitude of the slopes of parenting on self-efficacy at 2 SD below and above the mean of the personality dimension [50, 53, 57]. Third, further quantification of the interaction effect in terms of diathesis stress vs. differential susceptibility was conducted by computing the "proportion affected" (PA) index and the proportion of interaction index (POI), following recommendations of Roisman et al. [53]. The PA index identifies the proportion of participants who benefit from the positive environment. Evidence for the differential susceptibility hypothesis would come from a PA index around 0.50 and support for the diathesis-stress model from a PA index of 0.00 [53]. The PoI reflects the proportion of the total area in the interaction plot that can be attributed to differential susceptibility. Values of the PoI between 0.20 and 0.80 could be interpreted as supportive of the differential susceptibility hypothesis and values close to 0.00 suggest evidence for diathesis-stress [58, 59]. Finally, to test the possibility that differential susceptibility is an artifact of imposing a linear predictor model on a nonlinear diathesis-stress phenomenon, we tested three additional models that included (parenting)^2^, personality X (parenting)^2^ and both nonlinear effects. To provide evidence for differential susceptibility, results of these models must show that neither one of both quadratic terms, nor the combination of both is statistically significant [53].

The goodness-of-fit of the model was evaluated using the chi-square test, the Comparative Fit Index (CFI) and the Root Mean Square Error of Approximation (RMSEA). A non-significant chi-square, a CFI of above 0.95 [60] and a RMSEA of below 0.08 [61] indicate a good fit.

To test possible differences between mother and father parenting, model fit was compared for two multigroup models grouped by parenting informant (i.e., mother and father). In the first model, all specified paths were freely estimated. In the second model, all specified paths were constrained to be equal. To compare model fit, the log likelihood difference test was corrected for MLR estimation using the scaling correction factor [62].

## Results

### Descriptive statistics

Means, standard deviations and correlations among study variables are reported in Table 1. Childhood extraversion, emotional stability and imagination were positively related to self-efficacy in emerging adulthood. Self-reported parenting of mothers and fathers was not significantly related to self-efficacy. Most of the personality dimensions were negatively related to maternal and paternal overreactivity and positively related to maternal and paternal warmth.

**Table 1:** Pearson correlations, mean scores and standard deviations of all study variables

	1	2	3	4	5	6	7	8	9	10	*M*	*SD*
1. Age in years at time 1	–										10.78	1.13
2. Overreactive discipline (M)	.01	–									3.06	0.83
3. Overreactive discipline (F)	.01	.26***	–								3.16	0.82
4. Warmth (M)	.02	− .29***	− .10	–							4.24	0.42
5. Warmth (F)	− .10	− .18**	− .12*	.34***	–						3.64	0.57
6. Extraversion	− .11**	− .12*	− .21***	.26***	.19**	–					3.54	0.46
7. Benevolence	.09	− .37***	− .32***	.25***	.26***	.17**	–				3.54	0.43
8. Conscientiousness	− .04	− .20***	− .15**	.26***	.18**	.18**	.36***	–			3.38	0.56
9. Emotional Stability	.08	− .20***	− .24***	.17*	.11	.47***	.33***	.33***	–	–	3.48	0.56
10. Imagination	− .13*	− .18**	− .18**	.24***	.26***	.43***	.17**	.57***	.43***		3.79	0.50
11. Self-efficacy	.03	− .09	.04	.01	− .02	.19***	.002	.06	.28***	.19**	2.90	0.39

M, mother; F, father

*p < .05, **p < .01, ***p < .001

### Main effects of parenting and personality on self-efficacy

To examine main effects in the longitudinal prediction of self-efficacy in young adulthood (aim 1), we entered the covariates age and gender, five personality dimensions and two parenting variables as predictors of GSE (Fig. 1, model 1). First, a multigroup baseline unconstrained model was established against which a model that includes equality constraints was compared. In this constrained model, direct pathways from covariates and the seven predictors to self-efficacy were constrained to be equal across mother- and father-data. The fit of the baseline model could not be estimated because this was a saturated model (zero degrees of freedom). Model fit of the constrained model was (*χ*^2^ (9) = 22.87, *p* = 0.01, CFI = 0.77, RMSEA = 0.07 [0.03–0.10]). The Satorra-Bentler scaled chi-square was 22.87 (*df* = 9), *p* < 0.01, indicating that the model with equality constraints fitted the data significantly worse. Based on the modification indices, the effect of overreactivity on self-efficacy was freely estimated for mother and father ratings. This model indicated an adequate fit to the data (*χ*^2^ (8) = 5.74, *p* = 0.68, CFI = 1.00, RMSEA = 0.00 [0.00–0.05]). The Satorra-Bentler scaled chi-square was 5.73 (*df* = 8), *p* = 0.68, indicating that with the exception of overreactivity (mothers: *B* = − 0.03; *p* = 0.18, fathers: *B* = 0.03; *p* = 0.22), effects of the covariates and direct effects of personality and parenting on self-efficacy were similar for father and mother reports (Table 2). Model results showed that emotional stability was significantly related to more general self-efficacy eleven years later (b = 0.09, *p* < 0.001). Girls reported significantly lower self-efficacy than boys (b =  -0.08, *p* < 0.05). There were no significant main effects of parenting. This model explained 12% of the variance in self-efficacy.

**Table 2:** Model results for: main and interaction effects of parenting and personality on GSE

Self-efficacy	*B*	SE	95% CI for *B*		*p*
			LL	UL	
*Main effects (model 1)*					
Age	.013	.019	− .03	.05	.51
Gender	− .080*	.040	− .16	− .001	.047
Overreactivity	− .027 ^a^/.030 ^b^	.020/.025	− .07/− .02	.01/08	.18/.22
Warmth	− .019	.019	− .06	.02	.32
Extraversion	.030	.023	− .02	.08	.19
Benevolence	− .026	.024	− .07	.02	.28
Conscientiousness	− .017	.026	− .07	.03	.51
Emotional stability	.092	.026	.04	.14	< .001
Imagination	.045	.031	− .02	.11	.15
*Main effects (model 2)*					
Age	.015	.019	− .02	.05	.45
Gender	− .068	.040	− .15	.01	.09
Overreactivity	.000	.018	− .04	.04	.98
Warmth	− .014	.018	− .05	.02	.43
Extraversion	.030	.024	− .02	.08	.22
Benevolence	− .027	.023	− .07	.02	.23
Conscientiousness	− .015	.025	− .07	.04	.56
Emotional stability	.091**	.026	.04	.14	.001
Imagination	.040	.031	− .02	.10	.21
Extraversion × overreactivity	.018	.021	− .02	.06	.39
Extraversion × warmth	.002	.019	− .04	.04	.94
Benevolence × overreactivity	.050**	.017	.02	.08	.003
Benevolence × warmth	.021	.020	− .02	.06	.30
Conscientiousness × overreactivity	− .007	.023	− .05	.04	.77
Conscientiousness × warmth	− .033	.021	− .07	.01	.12
Emotional stability × overreactivity	− .040	.022	− .08	.004	.08
Emotional stability × warmth	.007	.022	− .04	.05	.75
Imagination × overreactivity	.015	.025	− .03	.07	.55
Imagination × warmth	.038	.028	− .02	.09	.18

*p < .05, **p < .01, ***p < .001

^a^Coefficient for mothers

^b^Coefficient for fathers

### Interaction effects of personality in relations between parenting and self-efficacy

To examine whether personality moderated the effect of parenting on GSE in line with the diathesis stress or the differential susceptibility hypothesis (aim 2), we included ten personality X parenting interactions in Model 2 (Fig. 1). First, we tested a model in which parameters for interaction effects were freely estimated. Next, a model was tested in which parameters for interaction effects were constrained across mothers and fathers. The constrained model indicated an adequate fit to the data (*χ*^2^ (10) = 9.04, *p* = 0.53, CFI = 1.00, RMSEA = 0.00 [0.00–0.06]). The Satorra-Bentler scaled Chi-Square was 9.04 (*df* = 10), *p* = 0.53, indicating that estimates of interaction effects were similar for father and mother reports of parenting. Finally, although the effect of overreactivity on self-efficacy was statistically different for mother and father ratings in Model 1, these effects of overreactivity were (again) non-significant for both parents in Model 2. Therefore, in order to obtain the most parsimonious model, we tested a fully constrained Model 2 in which all direct and interaction effects were constrained to be equal in mother and father data. Fit indices showed an adequate fit to the data (*χ*^2^ (19) = 20.38, *p* = 0.37, CFI = 0.98, RMSEA = 0.02 [0.00–0.05]). The Satorra-Bentler scaled Chi-Square was 20.38 (*df* = 19), *p* = 0.37, indicating that estimates were similar for father and mother reports of parenting. This most parsimonious model was selected as the final model.

**Fig. 2:**
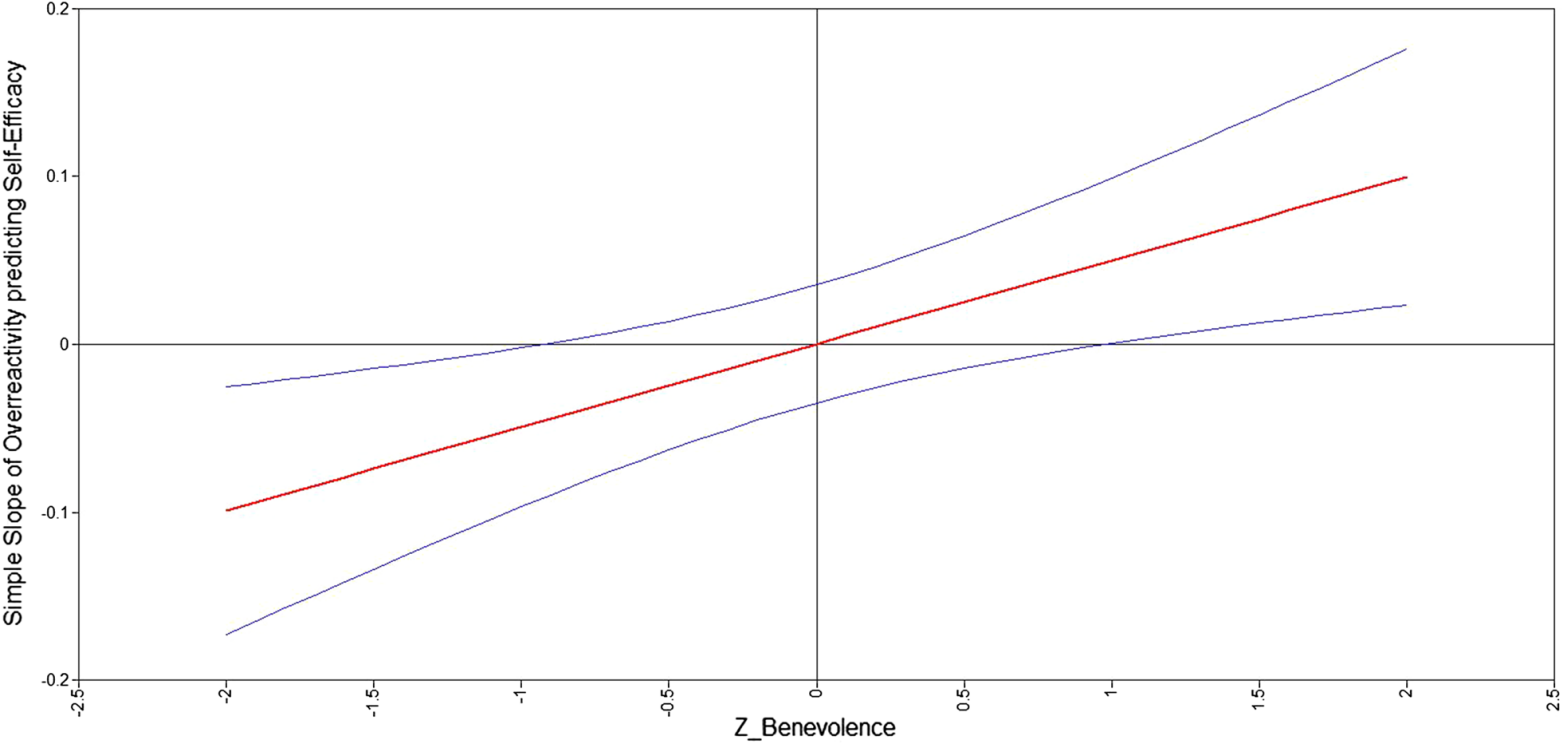
The association between overreactivity and self-efficacy at all levels of benevolence. *Note.* When benevolence is below − .09SD, higher levels of parental overreactivity are associated with lower the self-efficacy. When adolescents score higher than 1SD above the mean on benevolence, overreactivity is positively linked to self-efficacy

Model results of the moderator analyses (see Table 2) showed one significant interaction effect: children’s benevolence moderated the longitudinal effects of both mothers’ and fathers’ overreactivity on self-efficacy 11 years later. We plotted the region of significance for the slope of overreactivity on self-efficacy. As can be seen in Fig. 2, the effect of overreactivity on self-efficacy was not significant for children scoring between 0.9 SD below the mean, and 1.0 SD above the mean on benevolence. Among children scoring lower than − 0.9SD on benevolence (*n* = 110), as indicated by the area where the 95% confidence band did not contain the value of zero in Fig. 2, more overreactivity was associated with lower self-efficacy. Moreover, the strength of this effect increased when scores on benevolence decreased. Simple slope analyses showed that *B* (− 2SD) = − 0.10, [− 0.17 to − 0.02], *p* < 0.01. For children scoring higher than 1.0SD on benevolence (*n* = 94), more overreactivity was associated with higher self-efficacy and the strength of this effect increased when scores on benevolence increased. Simple slope analyses showed that *B*(2SD) = 0.10 [0.02–0.18], *p* = 0.01).

Further quantification of the interaction effect in terms of diathesis stress vs. differential susceptibility was conducted by computing the PA index and the POI (Roisman et al. [53]. In order to calculate the PA we identified the cross-over point, which was − 0.54 (− 0.027/0.050). The percentage in the sample that is differentially affected by the moderator, scoring lower than − 0.54 on overreactivity (standardized) was 32.1% of the mothers and 32.3% of the fathers in the sample, providing evidence for differential susceptibility. Next, the PoI was 0.25, which can be also be interpreted as evidence for differential susceptibility. Results of the three models with non-linear predictors indicated that none of the nonlinear predictors was significant whereas the interaction effect remained statistically significant. The final model with direct and interaction effects explained 14% of the variance in self-efficacy.

## Discussion

The current study was designed to enhance knowledge of individual and proximal contextual predictors of general self-efficacy (GSE) in early adulthood (20–23 years). In addition to examining main effects of children’s individual characteristics (Big Five personality traits; 9–12 years), and two types of parenting (warmth, overreactive discipline) by mothers and fathers on early adult GSE, we explored the extent to which children’s individual personality characteristics moderated effects of childhood parenting on early adult GSE. We tested whether moderation effects provided support for diathesis stress or the differential susceptibility models of person-environment interactions. Results indicated that child emotional stability was associated with higher GSE eleven years later, but main effects of parental warmth and overreactive discipline were non-significant. Further, we found evidence of one interaction effect showing that children’s benevolence functioned as a susceptibility marker in the longitudinal association between parental overreactivity and GSE. However, overall, the general lack of significant interaction effects is consistent with a main-effects only model.

### Main effects of childhood personality and parenting on early adulthood GSE

Regarding the main effects of mothers’ and fathers’ parenting and children’s personality traits, this study found that children’s emotional stability was associated with higher general self-efficacy eleven years later. That emotional stability is the most important predictor of self-efficacy is in line with the meta-analysis of Judge, Erez [31], examining associations among Big Five personality and general self-efficacy, and our study indicates that this holds across a long period of time. Emotional stability comprises, amongst others, the extent to which children are anxious (reverse-coded) and feel confident about themselves [46]. Our results suggest that this self-confidence in childhood may translate into feeling more efficacious about one’s own actions and capabilities in early adulthood, eleven years later. Children who score low on emotional stability are more insecure and cope less effectively with stressors [63] which may result in lower self-efficacy over time. Given that GSE is an important correlate of a wide range of (developmental) outcomes [5, 64, 65], knowing which individual characteristics are predictive of higher GSE may give direction to interventions aimed at improving early adult’s general self-efficacy. Results from this study suggest that targeting children’s feelings of anxiousness and self-confidence in interventions may have promising, long-term effects on their self-efficacy. Indeed, preliminary evidence for this hypothesis exists, with a 6-week school-based resilience intervention for 11–12-year-olds showing improvements in self-efficacy for all youth, and especially targeted the needs of children with negative affectivity [66]. Thus, especially for children low in emotional stability, such interventions might therefore in the long run improve their sense of self-efficacy.

Nevertheless, no other main effects of child personality, or of mothers’ and fathers’ parenting on early adults’ GSE were found to be significant. These mostly non-significant results are inconsistent with theory and empirical work examining short-term effects of child personality and parenting on child development [15, 22, 23]. The inconsistencies in the patterns of results may be due to the fact that the current study examined associations between constructs that were assessed more than a decade apart, and with a multi-informant design [i.e., different informants for the outcome (self-reports) versus predictor variables (parent-reports)]. That less significant longitudinal associations between personality characteristics and outcomes are found is in line with other work. Rioux, Castellanos-Ryan [67] for example reported statistically significant cross-sectional but no longitudinal relations between impulsivity and adolescence substance use.

### Moderation of parenting-GSE associations by personality

Second, we explored to what extent associations between mothers’ and fathers’ overreactive discipline and warmth and young adults’ GSE were moderated by children’s big five personality dimensions. We found evidence for one interaction effect, which supported the differential susceptibility model. Children with relatively low levels of benevolence were more sensitive to an adverse environment, with *high* mother- and father-reported overreactive discipline being associated to *less* GSE in early adulthood for these children only. Whereas, high benevolent children seem to be protected from the negative effects of high parental overreactivity, with *high* parental overreactivity being associated with *more* GSE for these children only. On the one hand, this result suggests that children scoring low on benevolence appear particularly vulnerable to the adverse effects of overreactive discipline, and these adverse effects may be long-lasting. This result is in line with earlier research focusing on interactions between parenting and personality with different outcome measures, for example aggression and rule-breaking trajectories [68], externalizing behavior [69], adolescent alcohol use [70] and trajectories of anxious and depressive problems [39]. Children scoring low on benevolence generally are more irritable and egocentric, and less compliant. The results indicate that these personal dispositions may form a vulnerability for these children in combination with overruling and strict discipline of parents. An additional possibility is that children who are more irritable and less compliant evoke more overreactive parenting, suggesting a mediating role of overreactivity or a dynamic and reciprocal process between children’s personality and parents resulting in more positive or negative developmental outcomes [42, 71].

In contrast, children scoring ‘average’ on benevolence appear not to be affected by mothers’ and fathers’ overreactive discipline in the long-term regarding their GSE. Rather surprisingly, children scoring high on benevolence reported higher GSE when their parents used more overreactive discipline and therefore seemed to be protected from the adverse effects of this parental practice. A possible explanation for this unexpected result is that children scoring high on benevolence are eager to obey their parents and that the strict discipline of their parents leads to more goal-driven behavior. This may result in higher levels of mastery and self-efficacy. Given that this is the first study to investigate long term effects of personality-parenting interactions and the inclusion of several interactions, future research should replicate this finding.

Consistent with our expectations, all main effects and interactions were similar (not statistically different) for (children of) mothers versus fathers. This pattern of results is in line with much research that employed stringent tests of parental gender differences [72, 73], and imply that generally, mothers and fathers are similarly important for their children’s outcomes.

### Limitations and future directions

Several limitations warrant caution in the interpretation of results. First, our study relied exclusively on questionnaire measures, which increases the risk of method bias. A related issue is the fact that parenting was only reported by the parents themselves. Levels of agreement between parent and child reports on parental behaviors are generally modest, with parents reporting more favorably than their children on parenting [74]. Therefore, a multimethod measurement strategy (e.g., the inclusion of observational measures) might result in more accurate assessment of parenting and children’s individual differences and thus further strengthen the results.

Second, our sample consisted solely of Western European families, and results of this study cannot be directly generalized to other samples. Previous studies have shown that the impact of parenting differs across cultural contexts. For example, overreactive discipline was found to be stronger related to childhood psychopathology in collective cultures than in individualistic cultures [75]. Future studies should examine the (main and interactive) effects of children’s individual personality characteristics and mothers’ and fathers’ parenting in samples with more diverse characteristics, not only in terms of ethnicity, but also, with regard to socio-economic status, family composition, and in families at-risk for child or parent psychopathology.

Third, our study is one of the first studies that examined prospective associations between childhood personality and parenting and emerging adulthood self-efficacy, through which we aimed to provide a more comprehensive picture of temporal associations. Nonetheless, it is generally considered to be ideal to also include the outcome measure at predictor time points. Such data could provide further insights into the directions of effects. Unfortunately, we only have self-efficacy available at one time point in emerging adulthood. In addition, parenting and personality interact with each other and shape each other at the same time [76]. Therefore, future research should also focus on the dynamic and reciprocal effects between parenting, child development, and children’s individual characteristics, preferably using measurements assessed at different time-scales (e.g., day-to-day, monthly, and yearly assessments) [77, 78]. Lastly, in a period of 11 years several life-events can occur that affect adjustment over time. Such so-called interim effects, unobserved events that can occur during the measured time period, might influence the outcome measurements [79]. On the other hand, despite such possible life-events that differ per person and that could affect self-efficacy, this study found significant associations between childhood personality and early adulthood general self-efficacy. Future research should focus on the effect of life-events and on mechanisms that can explain the prospective associations among personality and self-efficacy.

## Conclusion

The present study showed that childhood emotional stability is an important long-term predictor of self-efficacy, even into emerging adulthood. In addition, it was found that mothers’ and fathers’ overreactive discipline was related to self-efficacy eleven years later but only for children scoring either *low* or *high* on benevolence. Overall, results from our study suggest that child individual characteristics (emotional stability) may have long-lasting effects on children’s self-efficacy. Moreover, the integration of individual differences in models of parenting effects may further improve our understanding of early adults’ adjustment.

## Data Availability

The datasets used and/or analyzed during the current study are available from the corresponding author on reasonable request.

## Footnotes

1 Additional attrition analysis regarding attrition from the previous data waves to our selected T1 (available upon request from the last author) showed no meaningful differences.
